# Lipidomic Approaches to Study HDL Metabolism in Patients with Central Obesity Diagnosed with Metabolic Syndrome

**DOI:** 10.3390/ijms23126786

**Published:** 2022-06-17

**Authors:** Gabriele Mocciaro, Simona D’Amore, Benjamin Jenkins, Richard Kay, Antonio Murgia, Luis Vicente Herrera-Marcos, Stefanie Neun, Alice P. Sowton, Zoe Hall, Susana Alejandra Palma-Duran, Giuseppe Palasciano, Frank Reimann, Andrew Murray, Patrizia Suppressa, Carlo Sabbà, Antonio Moschetta, Albert Koulman, Julian L. Griffin, Michele Vacca

**Affiliations:** 1 Department of Biochemistry and Cambridge Systems Biology Centre, University of Cambridge, Cambridge CB2 1GA, UK; gm593@cantab.ac.uk (G.M.); antonio.murgia@owlstone.co.uk (A.M.); sn504@cam.ac.uk (S.N.); zoe.hall@imperial.ac.uk (Z.H.); 2 Department of Interdisciplinary Medicine, Clinica Medica “C. Frugoni”, Aldo Moro University of Bari, 70124 Bari, Italy; patrizia.suppressa@gmail.com (P.S.); carlo.sabba@uniba.it (C.S.); antonio.moschetta@uniba.it (A.M.); 3 Roger Williams Institute of Hepatology, Foundation for Liver Research, London SE5 9NT, UK; 4 Department of Medicine, University of Cambridge, Cambridge CB2 0QQ, UK; simodamo@hotmail.it; 5 Clinica Medica “A. Murri”, “Aldo Moro” University of Bari, 70124 Bari, Italy; palascianog44@gmail.com; 6 Welcome Trust-MRC Institute of Metabolic Science Metabolic Research Laboratories, Addenbrooke’s Hospital, Hills Road, Cambridge CB2 0QQ, UK; bjj25@medschl.cam.ac.uk (B.J.); rgk27@medschl.cam.ac.uk (R.K.); fr222@cam.ac.uk (F.R.); ak675@medschl.cam.ac.uk (A.K.); 7 Department of Biochemistry and Molecular and Cellular Biology, Veterinary Faculty, University of Zaragoza, 50013 Zaragoza, Spain; luis.herrera@imdea.org; 8 Department of Physiology, Development and Neuroscience, University of Cambridge, Cambridge CB2 3EG, UK; apb72@cam.ac.uk (A.P.S.); ajm267@cam.ac.uk (A.M.); 9 Biomolecular Medicine, Division of Systems Medicine, Department of Metabolism, Digestion and Reproduction, Imperial College London, London SW7 2AZ, UK; susana.palma-duran@crick.ac.uk; 10 Rowlett Institute, Foresterhill, University of Aberdeen, Aberdeen AB25 2ZD, UK

**Keywords:** obesity, lipoprotein metabolism, LC-MS, lipidomics, lecithin cholesterol acyltransferase (LCAT)

## Abstract

The metabolic syndrome (MetS) is a cluster of cardiovascular risk factors characterised by central obesity, atherogenic dyslipidaemia, and changes in the circulating lipidome; the underlying mechanisms that lead to this lipid remodelling have only been partially elucidated. This study used an integrated “omics” approach (untargeted whole serum lipidomics, targeted proteomics, and lipoprotein lipidomics) to study lipoprotein remodelling and HDL composition in subjects with central obesity diagnosed with MetS (*vs.* controls). Compared with healthy subjects, MetS patients showed higher free fatty acids, diglycerides, phosphatidylcholines, and triglycerides, particularly those enriched in products of *de novo* lipogenesis. On the other hand, the “lysophosphatidylcholines to phosphatidylcholines” and “cholesteryl ester to free cholesterol” ratios were reduced, pointing to a lower activity of lecithin cholesterol acyltransferase (LCAT) in MetS; LCAT activity (directly measured and predicted by lipidomic ratios) was positively correlated with high-density lipoprotein cholesterol (HDL-C) and negatively correlated with body mass index (BMI) and insulin resistance. Moreover, many phosphatidylcholines and sphingomyelins were significantly lower in the HDL of MetS patients and strongly correlated with BMI and clinical metabolic parameters. These results suggest that MetS is associated with an impairment of phospholipid metabolism in HDL, partially led by LCAT, and associated with obesity and underlying insulin resistance. This study proposes a candidate strategy to use integrated “omics” approaches to gain mechanistic insights into lipoprotein remodelling, thus deepening the knowledge regarding the molecular basis of the association between MetS and atherosclerosis.

## 1. Introduction

The metabolic syndrome (MetS) is a cluster of cardiovascular risk factors defined by the co-existence of central obesity, systemic hypertension, impaired glucose metabolism, and/or atherogenic dyslipidaemia (decreased high-density lipoprotein (HDL) and increased apolipoprotein (apo) B containing lipoproteins: very low-density lipoprotein (VLDL), and low-density lipoprotein (LDL)) [1]. As a result of the obesity epidemic, MetS has rapidly become a public health burden, with the prevalence in European countries estimated between 10% and 30% [2]. Furthermore, MetS leads to increased mortality due to its association with type 2 diabetes mellitus (T2DM), fatty liver, cardiovascular disease (CVD), and cancer [1,3]. MetS is a multifactorial condition whose pathophysiology is still under investigation. It has been demonstrated that obesity and insulin resistance (IR) profoundly reshape the lipoprotein profile and alter the quantity and quality of atherogenesis-affecting triglycerides (TG), cholesterol transport, and lipoprotein oxidation while promoting low-grade chronic inflammation [4,5]. Much less, however, is known about the contribution of other key structural lipids, such as phospholipids (PL), to this process [6].

The main circulating phospholipids phosphatidylcholines (PC) and lysophosphatidylcholines (LysoPC) are biochemically interconvertible species, and their metabolism can be modulated both intracellularly (e.g., in the liver, intestine, adipose tissue, or macrophages) and extracellularly. In the blood, PC can be remodelled into LysoPC by two enzymes: (i) lipoprotein-associated phospholipase A2 (Lp-PLA 2), an enzyme with pro- and anti-inflammatory capabilities [7] that catalyses the hydrolysis of fatty acids at the sn-2 position of oxidised phospholipids, ultimately generating a fatty acid and a LysoPC, and (ii) lecithin cholesterol acyltransferase (LCAT), a key player in the formation of large and mature HDL [8] that transfers 18:1 or 18:2 fatty acids from PC to free cholesterol (FC), thus generating cholesteryl esters (CE) and LysoPC [9]. Lipoprotein lipid remodelling is also ensured by (iii) phospholipid transfer protein (PLTP), which mediates the net transfer of PL from TG-rich lipoproteins into HDL particles alongside transferring several lipids, such as diglycerides, phosphatidic acids, sphingomyelins, cerebrosides, phosphatidylethanolamines, and α-tocopherol [10] (thus rendering this protein a non-specific transporter), and (iv) cholesteryl ester transfer protein (CETP), which is capable of transferring TG from ApoB lipoprotein to HDL in exchange for CE.

By thoroughly phenotyping lipid/lipoprotein metabolism, this study aims to expand the characterisation of lipid/HDL remodelling in early-stage and pharmacologically naïve MetS patients with central obesity.

Due to advances in mass spectrometry, it is now possible to characterise a plethora of circulating lipids with unprecedented throughput. Lipidomics has already proven to be a valuable tool in shedding light on metabolic disorders (including IR and non-alcoholic fatty liver disease) and cancer [11,12,13]. As recently highlighted in a systematic review of the literature, to date, only a few studies have investigated the lipidomic features of non-diabetic patients with MetS in whole serum [6], and even fewer have examined their HDL lipidome. Findings from the latter have been particularly challenging to harmonise because the pioneering studies investigated the major lipid classes (i.e., PL, CE, and TG) [14] with no information on single lipid species, while more recent studies differed in the way that lipids were normalised. Indeed, while Khan et al. reported the HDL lipidome as a function of HDL-PC content [15], Denimal et al. reported HDL lipidomic data as a percentage (mol%) of total lipids [16] along with the differences in the HDL subpopulation investigated. Further investigation is therefore warranted to understand how absolute changes in the HDL lipidome are associated with the disease and correlate with the cardio-metabolic risk.

Here, we employed state-of-the-art liquid chromatography coupled with mass spectrometry (LC-MS)-based lipidomics and proteomics, as well as lipoproteins lipidomics and biochemical analyses, to describe changes in HDL remodelling occurring in patients with central obesity and MetS. Overall, our results suggest that central obesity and MetS are associated with an impairment of HDL phospholipid metabolism and composition, which is partially driven by the lower activity of LCAT, with major changes correlating with the metabolic impairment of the patients.

## 2. Results

### 2.1. Clinical Characteristics of the Study Cohort

Compared with controls, MetS patients were older, showed higher body mass index (BMI) and waist circumference, alongside higher insulin resistance, as assessed by the Homeostasis Model Assessment 2 of Insulin Resistance (HOMA2-IR) (Supplementary Table S1). They also displayed mixed dyslipidaemia: remarkably higher TG and lower high-density lipoprotein cholesterol (HDL-C) (with an expected significant effect of sex on these parameters), alongside a higher ApoB-100/ApoA-1 ratio. MetS patients did not show significant differences in total cholesterol, low-density lipoprotein cholesterol (LDL-C), or blood pressure (BP) compared to control subjects (Supplementary Table S1). Regarding BP, 29% of the patients with increased BP were already receiving appropriate medical treatment.

### 2.2. Whole Serum Lipidomics of Healthy Participants and MetS Patients

**Figure 1:**
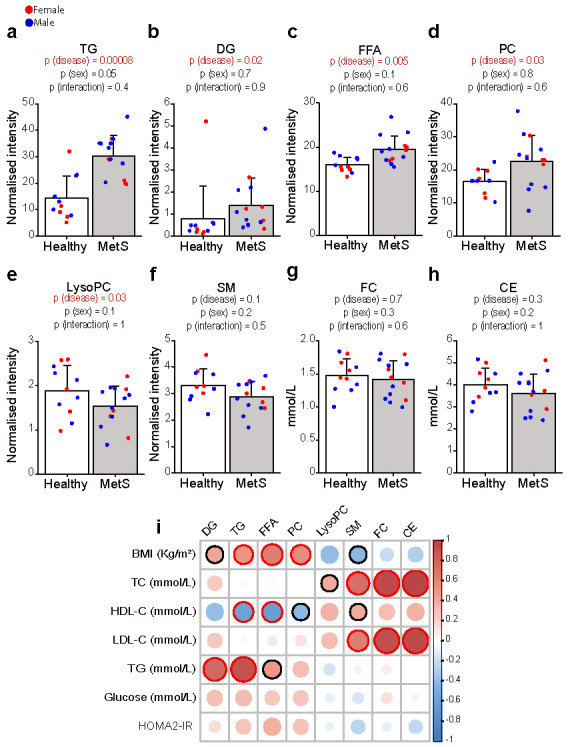
**The whole serum levels of major lipid classes in healthy and MetS participants.** TG (**a**), DG (**b**), FFA (**c**), and PC (**d**) were higher in MetS (*n* = 14), whereas LysoPC (**e**) were lower as compared with controls (*n* = 11). SM, FC, and CE (**f**–**h**) were not significantly different. Data are expressed as mean ± standard deviation. Statistical significance was assessed using two-way ANOVA using the disease state and sex as covariates; a *p*-value < 0.05 was considered significant. Detailed differences in the lipid species are described in Supplementary Figure S1 and Supplementary Table S2. (**i**) Heatmap representing a correlation matrix between metabolic features and lipidomic data in the whole cohort: colour represents the Pearson correlation coefficient (red: positive; blue: negative), and the size of the circle represents significance (red bold borders highlight correlations with *p* < 0.01; black bold borders highlight correlations with *p* < 0.05). All lipid species were analysed by liquid chromatography–mass spectrometry except for FC and CE, which were analysed colorimetrically, as reported in the method section. Abbreviations: TG, triglycerides; DG, diglycerides; FFA, free fatty acids; PC, phosphatidylcholines; LysoPC, lysophosphatidylcholines; SM, sphingomyelins; FC, free cholesterol; CE, cholesteryl esters; BMI, body mass index; TC, total cholesterol; HDL-C, high-density lipoprotein cholesterol; LDL-C, low-density lipoprotein cholesterol; HOMA2-IR, Homeostasis Model Assessment 2 of Insulin Resistance.

In keeping with the definition of MetS and the widely established literature pointing to hyperinsulinemia and adipose tissue insulin resistance (AT-IR) as drivers of MetS in overweight/obese subjects [17,18], patients with MetS showed higher levels of total TG, diglycerides (DG), and free fatty acids (FFA) (Figure 1a–c) as compared with controls. Not surprisingly, the most significant differences in TG were observed in those containing saturated and monounsaturated fatty acids (differences in single lipid species are detailed in Supplementary Figure S1 and Supplementary Table S2) that our group and others previously linked to *de novo* lipogenesis (DNL) [19,20,21]. However, MetS patients also displayed significantly higher levels of total PC along with lower LysoPC when compared with controls (Figure 1d–e), while total sphingomyelins (SM), CE, and FC (Figure 1f–h) did not differ between the two groups.

Some of the key drivers of the aforementioned differences were (1) lower levels of LysoPC containing polyunsaturated fatty acids (including LysoPC 20:5, 20:4, and 18:2); (2) lower abundance of CE 18:2; and (3) higher levels of different polyunsaturated PC (such as PC 40:9 and 42:9) in MetS compared to controls (Supplementary Table S2).

The correlation matrix between lipid classes and clinical data highlighted a negative correlation between HDL-C and PC and a positive correlation between LysoPC and HDL-C (Figure 1i). These results point to a possible impairment in phospholipid lipoprotein metabolism occurring in MetS, associated with the severity of the metabolic impairment and possibly secondary to imbalances in HDL formation and/or remodelling.

### 2.3. Whole Serum Apolipoprotein Profile of Healthy Participants and MetS Patients

**Figure 2:**
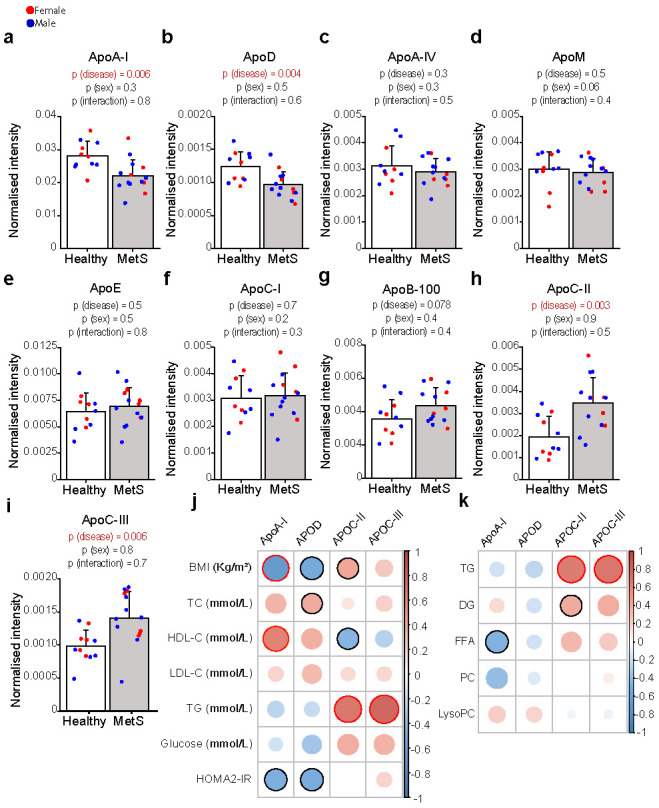
**Serum levels of major apolipoproteins in healthy and MetS participants.** In MetS (*n* = 14), ApoA-I (**a**) and ApoD (**b**) were lower, ApoA-IV, ApoM, ApoE, ApoC-I and ApoB-100 (**c**–**g**) were not significantly different, whereas ApoC-II (**h**) and ApoC-III (**i**) were higher as compared with controls (*n* = 11). All apolipoproteins were analysed by liquid chromatography–mass spectrometry, as reported in the method section. Statistical significance was assessed using two-way ANOVA using the disease state and sex as covariates; a *p*-value < 0.05 was considered significant. Data are mean ± standard deviation. (**j**) Heatmap representing a correlation matrix among significantly different apolipoproteins and metabolic features in MetS and healthy controls: colour represents the Pearson correlation coefficient (red: positive; blue: negative), and the size of the circle represents significance (red bold borders highlight correlations with *p* < 0.01; black bold borders highlight correlations with *p* < 0.05). (**k**) Heatmap representing a correlation matrix among significantly different lipidomic and apolipoprotein species in MetS and healthy controls: colour represents the Pearson correlation coefficient (red: positive; blue: negative), and the size of the circle represents significance (red bold borders highlight correlations with *p* < 0.01; black bold borders highlight correlations with *p* < 0.05). Abbreviations: ApoA-I, apolipoprotein A-I; ApoD, apolipoprotein D; ApoA-IV, apolipoprotein A-IV; ApoM, apolipoprotein M; ApoE, apolipoprotein E; ApoC-I, apolipoprotein C-I; ApoB-100, apolipoprotein B-100; ApoC-II, apolipoprotein C-II; ApoC-III, apolipoprotein C-III; BMI, body mass index; TC total cholesterol; HDL-C, high-density lipoprotein cholesterol; LDL-C, low-density lipoprotein cholesterol; HOMA2-IR, Homeostasis Model Assessment 2 of Insulin Resistance.

As apolipoproteins are key regulators of circulating lipid metabolism we studied the apolipoprotein profile of our cohort. In agreement with the low levels of HDL-C, MetS patients had significantly lower levels of ApoA-I and ApoD (Figure 2a,b), while ApoA-IV, ApoM, ApoE, ApoC-I, and ApoB-100 did not show significant differences between groups (Figure 2c–g). On the other hand, ApoC-II and ApoC-III were remarkably higher in MetS patients as compared with controls (Figure 2h,i). High levels of ApoC-III are known to contribute to hypertriglyceridemia via the inhibition of (a) the activity of lipoprotein lipases (LPL) and (b) the uptake of TG by the liver [22], thus resulting in an increased half-life of TG-rich lipoproteins; elevated levels of ApoC-II have already been reported in patients with T2DM and obesity [23,24]; however, the biological meaning of differences in ApoC-II levels is yet to be fully elucidated, as this apolipoprotein seems to have LPL-promoting activity [25]. The correlation analysis (Figure 2j) confirmed the expected positive correlations of ApoA-I with HDL-C (while being negatively correlated with BMI and HOMA2-IR) and of ApoC-II/ApoC-III with TG. Moreover, we also found interesting correlations between lipidomic and proteomic data (Figure 2k): in addition to the expected positive correlations between TG/DG and ApoC-II/ApoC-III (in light of the biological functions of these lipoproteins), PC and LysoPC positively correlated with ApoA-I, thus further suggesting that differences in whole serum phospholipid concentrations could be due to modifications in HDL composition.

### 2.4. LCAT, but Not Lp-PLA2, Activity Is Reduced in MetS

Apart from the anticipated lipidomic and apolipoprotein features characterising the MetS, our results point to the remodelling of the circulating lipidome. To gain insights into the biology of lipoprotein’s remodelling, we determined the activity levels of circulating lipoprotein remodelling enzymes.

While Lp-PLA2 and CETP were not significantly different between the two groups (data not shown), PLTP was significantly higher in MetS compared with controls (Supplementary Figure S2).

**Figure 3:**
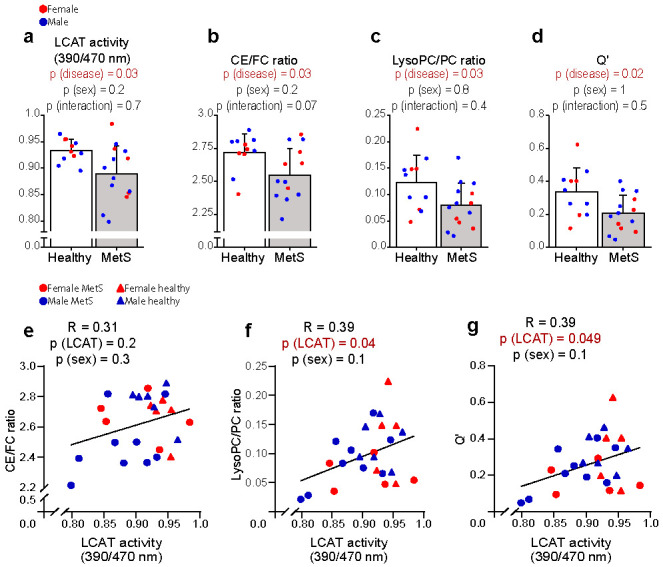
**Direct and indirect assessment of LCAT activity in healthy and MetSparticipants.** The direct measurement of LCAT activity (**a**) and its lipidomic predictors (**b**–**d**) were significantly lower in MetS (*n* = 14) when compared with controls (*n* = 11). The latter (**e**–**g**) positively correlated with LCAT activity. All lipid species were analysed by liquid chromatography–mass spectrometry except for FC and CE, which were analysed colorimetrically, as reported in the method section. LCAT activity was measured with a fluorometric assay in kinetic measurements. Statistical significance was assessed using two-way ANOVA using the disease state and sex as covariates; a *p*-value < 0.05 was considered significant. Data are mean ± standard deviation. The correlation between variables was assessed with Pearson’s correlation coefficient; the statistical significance was calculated using multiple regression to control for the effect of sex; a *p*-value < 0.05 was considered significant. Abbreviations: LCAT, lecithin–cholesterol acyltransferase; CE, cholesteryl esters; FC, free cholesterol; LysoPC, lysophosphatidylcholines; PC, phosphatidylcholines; Q’, (CE*LysoPC)/(FC*PC).

As our group recently showed in an independent cohort of patients with increased visceral obesity [26], we found LCAT activity to be significantly lower in the MetS group as compared with controls (Figure 3a). Here, by assessing different product/substrate ratios (proposed as indirect proxies of LCAT activity [27,28,29]) using lipidomics, we went a step further in showing that the lower LCAT activity has functional implications in the lipidome. While the LysoPC/PC ratio can be affected by both LCAT and Lp-PLA2 activity (the latter not being different in our cohort), the conversion of FC to CE in plasma can only be catalysed by LCAT [30]. As shown in Figure 3b,c, both CE/FC and LysoPC/PC ratios were significantly lower in MetS patients. This was even more evident (Figure 3d) when combining the information from cholesterol and phospholipids using a recently proposed formula, called the “non-equilibrium reaction quotient” (Q’; formula: (CE*LysoPC)/(FC*PC)) [27]. Furthermore, in support of the notion that the differences in the aforementioned lipid species (product/substrate ratios) reflect LCAT activity, we found significant positive correlations amongst these indirect proxies and the enzymatic assay (Figure 3e–g).

Taken together, these data suggest that the differences in the whole serum lipidome likely reflect, at least in part, suppressed LCAT activity.

### 2.5. LCAT Activity and Its Lipidomic Proxies Show an Inverse Correlation with Metabolic Risk Factors and Positively Correlate with HDL-C

The role of LCAT in the pathophysiology of atherosclerosis is highly debated, with conflicting results coming from pre-clinical and clinical studies [30]. Contradictory findings have also been reported in patients with metabolic risk factors [26,31,32,33,34], further highlighting the intricate nature of the LCAT function. Because of the coherent reduction in LCAT activity and its product-to-substrate ratios in MetS, we sought to understand the extent to which these parameters correlated with characteristic metabolic risk factors linked to MetS, such as BMI, HOMA2-IR, and HDL-C.

**Figure 4:**
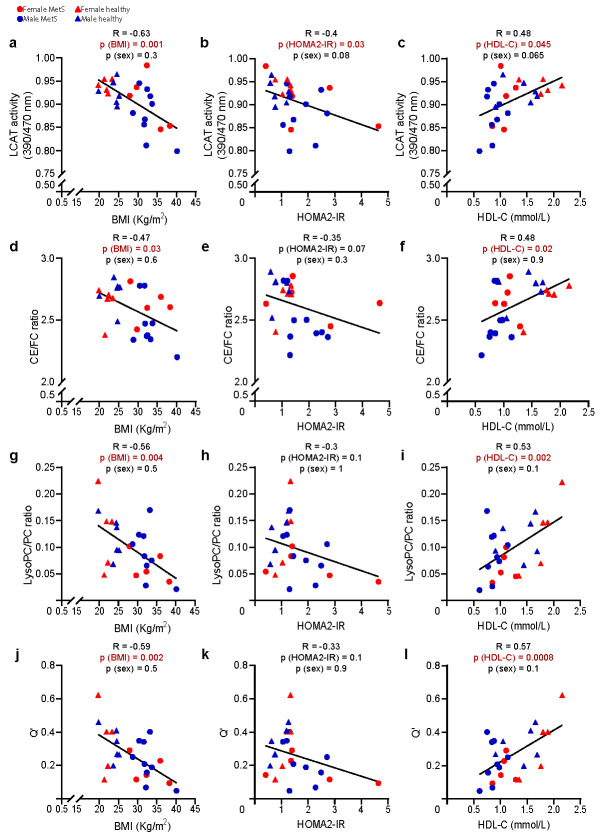
**Correlation between direct/indirect measurements of LCAT activity and metabolic risk factors.** Lipidomic predictors and direct measurement of LCAT activity (**a**–**l**) were negatively correlated with BMI and HOMA2-IR and positively correlated with HDL-C. All measurements were performed on MetS (*n* = 14) and controls (*n* = 11). Lipid species were analysed by liquid chromatography–mass spectrometry except for FC and CE, which were analysed colorimetrically, as reported in the method section. The correlation between variables was assessed with Pearson’s correlation coefficient; the statistical significance was calculated using multiple regression to control for the effect of sex; a *p*-value < 0.05 was considered significant. Abbreviations: LCAT, lecithin–cholesterol acyltransferase; CE, cholesteryl esters; FC, free cholesterol; LysoPC, lysophosphatidylcholines; PC, phosphatidylcholines; Q’, (CE*LysoPC)/(FC*PC); BMI, body mass index; HDL-C, high-density lipoprotein cholesterol; HOMA2-IR, Homeostasis Model Assessment 2 of Insulin Resistance.

LCAT activity and/or all of the lipidomic indices predicting its activity showed remarkable negative correlations with BMI (Figure 4a,d,g,j) and HOMA2-IR (Figure 4b,e,h,k) and positive correlations with HDL-C (Figure 4c,f,i,l). Q’ showed the highest correlation (R = 0.57) and significance (*p* = 0.0008) with HDL-C, further strengthening our hypothesis that the observed lipidomic differences were driven by HDL remodelling, which might go beyond the lower HDL cholesterol content that has been well described in MetS.

Taken together, these data suggest that LCAT activity and its lipidomic proxies display a strong relationship with cardio-metabolic risk factors, this being partially attributable to lower HDL-C levels in the MetS group compared with controls. Moreover, together with an impairment of cholesterol efflux (associated with reduced ABCA1/G1 monocyte/macrophage expression) that we and others reported in MetS [35,36], these data point to a possible impairment in HDL metabolism at multiple levels, which might affect not only HDL concentration and function (reverse cholesterol transport, RCT) but also the composition of HDL lipids.

### 2.6. HDL Composition in Central Obesity and MetS

**Figure 5:**
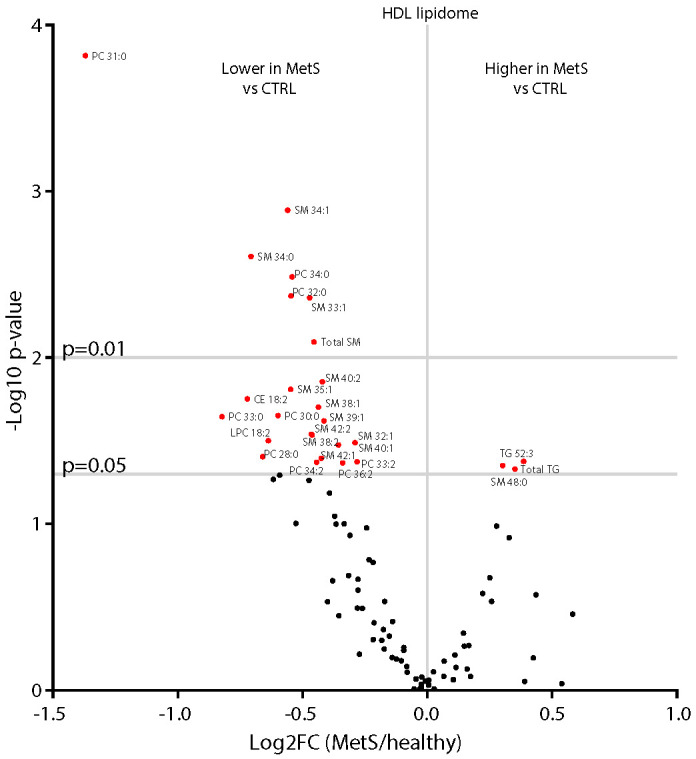
**Differences in the HDL lipidome in healthy and MetS participants.** The HDL lipidome of MetS participants (*n* = 14) showed lower levels of specific sphingomyelins (SM), phosphatidylcholines (PC), lysophosphatidylcholine (LysoPC) 18:2, and cholesteryl ester (CE) 18:2 and higher total triglycerides (TG) along with TG 52:3 and SM 48:0 when compared with healthy subjects (*n* = 11). Statistical significance was assessed by Student’s two-sided T-Test; a *p*-value < 0.05 was considered significant. Lipid species were analysed by liquid chromatography–mass spectrometry, as reported in the method section. The mean values of each lipid in healthy and MetS participants, along with unadjusted and adjusted false discovery rates (FDRs) and *p*-values, are reported in Supplementary Table S5.

**Figure 6:**
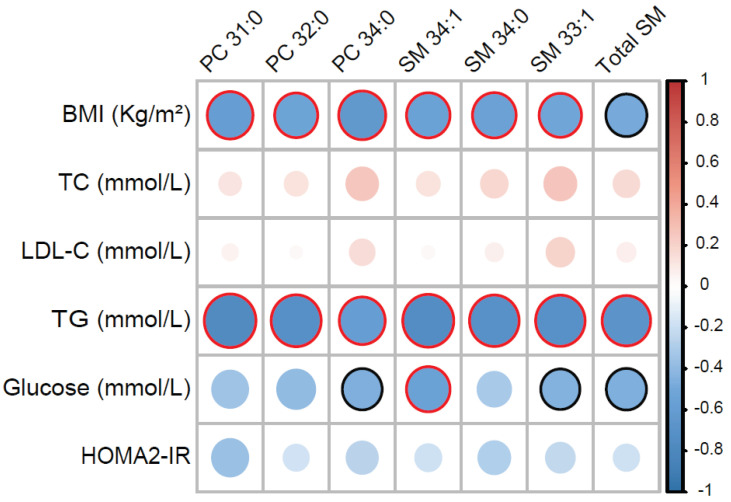
**HDL lipid species correlate with clinical features of metabolic impairment.** Heatmap representing a correlation matrix among highly significantly different HDL lipid species (*p* < 0.01) and metabolic parameters in MetS (*n* = 14) and healthy controls (*n* = 11): colour represents the Pearson correlation coefficient (red: positive; blue: negative), and the size of the circle represents significance (red bold borders highlight correlations with *p* < 0.01; black bold borders highlight correlations with *p* < 0.05). Abbreviations: PC, phosphatidylcholines; SM, sphingomyelins; BMI, body mass index; TC total cholesterol; LDL-C, low-density lipoprotein cholesterol; TG, triglycerides; HOMA2-IR, Homeostasis Model Assessment 2 of Insulin Resistance.

Lastly, to gain insights into the functional implications in terms of HDL composition led by the impairment in HDL formation [26,37] and remodelling, we characterised the differences in the HDL lipidome occurring in MetS. As shown in Figure 5, when compared with those of controls, MetS HDL showed significantly higher total TG and saturated very long chain SM (SM 48:0). Increased HDL-TG reduce HDL stability, rendering them more prone to the dissociation of ApoA-I and thus potentially increasing their catabolism [38]. On the other hand, several lipids belonging to PC, LysoPC, CE, and SM showed lower abundance in MetS compared with controls (Figure 5). Specifically, within HDL, both the product and substrates of LCAT (i.e., CE 18:2, LPC 18:2, and PC 34:2) alongside several MUFA SM were lower in MetS compared with controls. Reduced levels of PC and SM within HDL have been associated with the decreased efflux capacity of HDL particles, both in vitro and in observational studies [39,40,41,42,43]. Moreover, reduced PC and SM in HDL have been reported in patients with CVD [44], while the data in MetS cohorts are more debated [15,16]. The cluster of PC and SM that were identified as being most significantly reduced (*p* < 0.01) in MetS compared with controls also correlated negatively with BMI, TG, and glycaemia (Figure 6), therefore highlighting their importance as targets of future mechanistic studies to better characterise their biological importance.

The presence of odd-chain fatty acids among some of the most significantly reduced lipids within the HDL fractions suggests a potentially novel role for HDL as a carrier of health-promoting lipids, as odd-chain fatty acids have been previously associated with a reduced incidence of T2DM [45,46]. The provenience of these lipids requires further investigation, as dietary sources [47], gut microbiota [48], alpha-oxidation [49], and mitochondrial catabolism of BCAA [50] can potentially contribute to the pool of circulating odd-chain fatty acids.

Overall, our data highlight that whole serum lipidomics is highly influenced by lipoprotein’s metabolism and can provide useful insights into these processes; however, lipoproteins lipidomics offers a more detailed picture of the biology underlying systemic lipid metabolism.

## 3. Discussion

Central obesity and MetS are characterised by mixed dyslipidaemia: its treatment is a cornerstone for primary and secondary prevention of CVD. Beyond the traditional lipid markers (e.g., HDL-C and TG), several studies have characterised the lipidomic signature of abdominal obesity and MetS, but knowledge around the biological meaning of these signatures in disease and the underlying mechanisms remains limited.

In this study, we were able to confirm multiple previously described MetS-associated lipidomic changes related to IR and hyperinsulinemia (DG and TG, specifically enriched in DNL products) [6,19] and to describe novel findings that correlate with the metabolic burden of central obesity and MetS.

The phospho-lipidomic signature of MetS patients is more debated in the literature, probably because it is more influenced by differences in study design (recruitment criteria, normalisation of lipid species, lipids detected, and lipoprotein separation method). We confirmed higher levels of total PC in MetS vs. controls, as shown previously by others [51,52,53], although conflicting results have also been published [54,55]. We considered the higher levels of PC in MetS vs. controls unexpected, considering that, at least in healthy subjects, PCs are predominantly contained in HDL particles [56] and that HDL-C and ApoA-1 were significantly lower in our cohort of MetS participants.

Investigating HDL composition in more detail, we were indeed able to show that HDL-PC concentrations are lower, as expected, and this correlated with cardio-metabolic risk factors. While reduced SM levels within HDL are associated with decreased RCT (in vitro), some in vitro and pre-clinical studies showed an inhibitory effect of SM on LCAT [57]. This suggests that, in MetS, other pathways (e.g., reduced ApoA-1) might explain the lower LCAT activity levels but not HDL-SM levels. A deeper look into HDL lipidomics therefore suggested that (1) whole serum lipidomics can be misleading when lipoprotein fractions are under metabolic pressure, altering lipid physiology; (2) differentially studying the lipid composition of lipoprotein fractions can be more informative in cardio-metabolic studies than whole serum lipidomics, also clarifying some apparent conflicting results; (3) there is an urgent need to deepen the knowledge of PL metabolism and function, as it might help to better understand the role of HDL in cardio-metabolic disease; and (4) last, a consensus in lipoprotein lipidomic data analysis is currently needed: we suggest that apparently conflicting results in the literature are partly due to the different methods used to isolate the lipoproteins and process the data (lipid normalisation) [14,15,16,58]. Indeed, the expression of data as percentages is very common in lipidomic studies; however, because this normalisation is influenced by the abundance of other lipids (and different laboratories have different arrays of lipids detected by their assays), the use of absolute values should be preferred, as recommended by a recent position paper for the standardisation of lipidomic human blood samples [59].

Another result that attracted our attention was the differential regulation between LysoPC (lower) and PC (higher) in whole serum lipidomics: since PC and LysoPC are biochemically interconvertible species, we checked if our data could help to study disease-associated changes in the activity of lipid-remodelling enzymes. Here, we described a reduction in LCAT activity in patients with central obesity and MetS that leads to functional PL/cholesterol remodelling, and we investigated the association of multiple lipidomic ratios with standard enzymatic activity assays as valuable predictors of LCAT activity (as confirmed by a strong correlation among direct/indirect measurements). Moreover, Q’ has been shown to be reduced in patients with CVD [27]: intriguingly, assessing LCAT activity either directly or with lipidomic ratios, we found that it was reduced in our early-stage naïve MetS patients with mild cardiovascular risk and without organ damage; and correlated with metabolic features: these findings therefore suggest that reduced LCAT activity is an early event associated with central obesity and insulin resistance, and that lipidomic ratios might serve in the future as potential predictive biomarkers of HDL function and/or cardiovascular risk.

Last, although it was beyond the scope of this work to describe the mechanisms by which LCAT activity was reduced, the proteomics data allow us to speculate that contributors to the reduced activity might be the lower levels of ApoA-I (the main activator of LCAT) [30] and elevated ApoC-III levels (an inhibitor of LCAT) [60]. These data coherently point toward reduced LCAT activity in our cohort. Reduced LCAT activity, together with reduced cholesterol efflux and lipoprotein oxidation [61], might contribute to the close association between MetS, impaired RCT, and atherosclerosis. However, it is important to underline that, although the crucial role of LCAT in the maturation of HDL has been elucidated by intensive investigation over the last 50 years, its role in CVD is still debated [62], and larger studies are needed to stratify patients against cardio-metabolic risk.

This study has some limitations. First, we used data from the highest number of available participants: although multiple layers of evidence support our findings, non-significant results must be interpreted with caution in light of the small sample size (all variables with an effect size < 1.1 were not sufficiently powered in the post hoc power analysis). Second, this study might suffer from a bias of recruitment: the presence of atherogenic dyslipidaemia (high TG; low HDL-C) is one of the MetS-defining criteria [63], potentially explaining some differences in the lipidomic results shown by others. Third, the Q index does not account for endothelial and hepatic lipase activities, which are able to remodel circulating phospholipids; however, the robust correlation with LCAT activity reassures that most of the changes in the Q index observed in MetS were due to LCAT activity per se. Fourth, while LCAT is the only enzyme capable of converting FC to CE in circulating blood, their ratio might be impacted by the altered transfer of FC from peripheral tissues to lipoproteins [64]. Last, our results (despite finding multiple lines of validation in the literature [18,19,26,27,44,65,66]) will require validation in a larger study cohort to prove the transferability of our main findings to clinical practice.

In conclusion, our results suggest that central obesity and MetS are associated with an impairment of phospholipid remodelling and HDL composition, partially led by reduced LCAT activity and correlating with obesity and insulin resistance. This study also suggests the need for a candidate strategy to use integrated lipidomic and proteomic approaches to gain mechanistic insights into lipoprotein remodelling in order to deepen the knowledge regarding the molecular basis of the association between MetS and atherosclerosis.

## 4. Materials and Methods

### 4.1. Ethics and the MetS Study Cohort

Eleven healthy volunteers and fourteen patients with central obesity, at the first diagnosis of MetS, with no evidence of organ damage, were included in this study. The presence of MetS was defined when at least 3 (out of 5) criteria for MetS were present according to the National Cholesterol Education Program Third Adult Treatment Panel (ATP III) [63]: all (100%) patients showed increased WC and were overweight/obese (21% had ≥ 25 BMI < 30; 79% had BMI ≥ 30). The other MetS criteria were: reduced HDL-C (93%); increased glucose (57%); increased triglycerides (42%); and increased BP (or in treatment for hypertension—43%). Exclusion criteria were: the presence of diseases that could have influenced participants’ metabolism (i.e., autoimmune disease, cancer, endocrine disorders, and acute and chronic kidney failure), smoking, alcohol intake of over 25 g/day, and pharmacological treatment (hypertension drugs in MetS patients were allowed). None of the participants had documented significant atherosclerosis at the carotid Doppler ultrasound; also, the average intima-media thickness, a marker of subclinical atherosclerosis, of the MetS group was within the normal range (data not shown). The clinical characteristics of the study population are summarised in Supplementary Table S1.

### 4.2. Sample Collection and Clinical Biochemistry Measurements

After overnight fasting, serum was collected from healthy and MetS participants for the assessment of standard clinical biochemistry tests. Serum was separated by centrifugation and stored at −80 °C. Clinical biochemistry, including the full lipid profile, glucose, insulin, and circulating liver enzymes, was performed by The Pathology Partnership (Addenbrooke’s Hospital, Cambridge, UK). Homeostasis Model Assessment 2 (HOMA2-IR) values were calculated using the HOMA Calculator v2.2.3 available at https://www.dtu.ox.ac.uk/homacalculator/ (accessed on 5 May 2019).

### 4.3. HDL Isolation

The serum HDL separation was performed by size-exclusion chromatography (SEC) using a previously described method [67]. Briefly, fractions were determined in 50 μL of serum samples diluted with 50 μL of a PBS solution via SEC, using a Superose 6 increase column (10/300GL, 10 × 300, 24 mL), (GE Healthcare; Uppsala, Sweden). SEC was carried out using an ÄKTA purifier 10 (GE Healthcare; Uppsala, Sweden), equipped with a fraction collector (Frac-950). The system was controlled by a UNICORN control system, version 4.10 (GE Healthcare; Uppsala, Sweden). The SEC flow rate was set at 450 μL/min. Eluting fractions were collected in glass-coated 96-well plates (Eppendorf Protein Low-Bind; Hamburg, Germany). The HDL fractions were combined after fractionation, and samples were dried in a vacuum centrifuge and stored at −80 °C before further processing. Lipids from the selected fractions obtained by Superose-6 SEC were further extracted and investigated by LC-MS, as detailed below.

### 4.4. Lipid Extraction for Mass Spectrometry Analyses

Lipids were extracted from blood serum and dried HDL fractions, previously stored at −80 °C, using an adaptation of the Folch method [68]. Briefly, 10 μL of blood serum was mixed with chloroform/methanol (2:1, 1 mL), and then 10 μL of internal standard mix including the following: cholesteryl-d7 pentadecanoate (15:0d7 CE), heptadecanoic-d33 acid (17:0-d33 FFA), 1-palmitoyl(D31)-2-oleyl-sn-glycero-3-phosphatidylcholine (16:0-d31-18:1 PC), N-palmitoyl(d31)-d-erythro-sphingosylphosphorylcholine (16:0-d31 SM), and glyceryl tri(pentadecanoate-d29) (45:0-d87 TAG) (Avanti Polar Lipids Inc, Alabaster, AL, USA) was added. Samples were sonicated for 10 min, and subsequently, deionised water was added (400 μL). Samples were then centrifuged (13,000× *g*, 10 min). The organic fraction was transferred into a new vial and dried under a stream of nitrogen. Dried samples were then stored at −80 °C until analysis.

### 4.5. Lipid Analysis by LC-MS

Before analysis, dried samples were reconstituted in 20 μL of 1:1 chloroform/methanol, sonicated for 10 min, and then diluted in isopropyl alcohol/acetonitrile/water (2:1:1, 100 μL). The analysis of intact lipids was performed through LC-MS using a Dionex Ultimate 3000 ultrahigh-performance liquid chromatography system (UHPLC; Thermo Scientific, Hemel Hempstead, UK) coupled to an LTQ Orbitrap Elite Mass Spectrometer (Thermo Scientific, Hemel Hempstead, UK). A 10 μL sample was injected onto a C18 CSH column, 1.7 μm pore size, 2.1 mm × 50 mm (Waters Ltd., Manchester, UK), maintained at 55 °C. A gradient of solvent A, 10 mM ammonium formate in acetonitrile/water 6:4, and solvent B, 10 mM ammonium formate in isopropanol/acetonitrile 9:1, was used for the positive mode acquisition. For the negative mode, the mobile phase remained the same except for the use of 10 mM ammonium acetate instead of ammonium formate. HPLC was coupled to a heated electrospray source held at 365 °C. The data were collected in positive and negative ion modes with a mass range of 100–2000 *m/z*. Peaks in the spectra were detected and integrated by XCMS software with an in-house script running under R (version 4.0.0). Lipid peak areas were normalised (divided) to their class-specific stable isotopically labelled internal standard peak areas, leading to semi-quantitative data, here referred to as normalised intensity. With regard to missing values, lipids with missing values higher than 30% were removed from the dataset, whereas the others were imputed with half of the minimum value detected for that specific lipid [69].

### 4.6. Esterified Cholesterol Analysis by LC-MS/MS

The organic lipid-containing layer from the Folch extraction was analysed by targeted LC-MS/MS using a UHPLC+ series coupled to a TSQ Quantiva mass spectrometer (Thermo Fisher scientific, Waltham, MA, USA). Ten microlitres of sample containing isotopically labelled cholesteryl-d7 pentadecanoate (Avanti Polar Lipids Inc, Alabaster, AL, USA) was injected onto an Acquity C18 CSH column (Waters Ltd., Warrington, UK; 100 × 2.1 mm, 1.7 μm) with a column temperature of 45 °C. A gradient separation was used as described for the lipidomics experiments. A heated electrospray ionisation source was operated in positive ion mode; desolvation temperature and gas flow were 270 °C and 45 arbitrary units, respectively. The selected reaction monitoring transitions used are included in Supplementary Table S3. Xcalibur software (Thermo Fisher Scientific, Hemel Hempstead, UK)) was used for peak integration. Peak areas of whole serum were normalised to the internal standard, while HDL lipids were further normalised by the total protein content of the HDL fraction (Pierce bicinchoninic acid assay).

### 4.7. HDL Total Protein Concentration

Total protein concentration in the HDL fraction was determined by bicinchoninic acid assay (Pierce BCA; Thermo Fisher Scientific, Waltham, MA, USA) according to the manufacturer’s instructions.

### 4.8. Whole Serum Protein Digestion for LC-MS/MS Analysis

Serum (10 μL) was diluted by a factor of 10 with 50 mM ammonium bicarbonate in water. Diluted serum (10 μL) was then transferred to 490 μL of 10 mM dithiothreitol in 50 mM ammonium bicarbonate, with 0.5 mg/mL bovine serum albumin (BSA) as an internal standard. Samples were incubated at 60 °C for 1 h and allowed to cool to room temperature, after which 100 μL of 100 mM iodoacetamide in 50 mM ammonium bicarbonate was added and samples were incubated at room temperature in the dark for 30 min. Trypsin (10 μL at 100 μg/mL) was added to a 100 μL aliquot of each sample and incubated for 16 h at 37 °C. After incubation, 20 μL of 1% formic acid was added to halt digestion prior to analysis by LC-MS/MS.

### 4.9. Apolipoprotein Analysis by LC-MS/MS

LC-MS/MS analysis was performed on an M-Class liquid chromatography system coupled to a XevoTQ-XS triple quadrupole (Waters, Milford, MA, USA). A gradient of solvent A, 0.1% formic acid in water, and solvent B, 0.1% formic acid in acetonitrile, was used to separate digested proteins. The sample (2 µL) was injected onto an HSS T3 50 × 1.0 mm column (Waters) at 25 µL per minute at 15% B, rising to 50% B over 5 min. The column was washed with 85% B for 2.5 min before returning to initial conditions for a total run time of 10 min. Positive electrospray ionisation was performed with a needle voltage of 3 kV, source and desolvation temperatures of 150 °C and 450 °C, respectively, and a cone voltage of 30 V. The selected reaction monitoring transitions used are included in Supplementary Table S4. Peptide peaks were integrated using TargetLynxXS (Waters, Milford, MA, USA) and expressed as a ratio of the average peak area to the mean peak area value of two typically digested BSA peptides.

### 4.10. Lipoprotein-Associated Phospholipase A2 (Lp-PLA2) Activity

Lp-PLA2 activity in serum was measured in duplicate using a commercially available kit (Cayman, Europe) following the manufacturer’s instructions. Specifically, samples were incubated for 30 min with Ellman’s reagent at room temperature with the subsequent addition of 2-thio PAF, used as a substrate for Lp-PLA2 activity. The reaction causes an increase in colourimetric absorbance, which was measured once every minute at 405–414 nm. Measurements were obtained using a plate reader (Tecan Infinite 200 PRO) (Tecan, Mannedorf, Switzerland).

### 4.11. Phospholipid Transfer Protein (PLTP) Activity

PLTP activity in serum was measured in duplicate using a commercially available kit (Merck, St. Louis, MO, USA) following the manufacturer’s instructions. Specifically, the assay uses a proprietary substrate to detect PLTP-mediated transfer of the fluorescent substrate. The transfer activity results in an increase in fluorescence intensity at 465/535 nm. Measurements were obtained using a plate reader (Tecan Infinite 200 PRO) (Tecan, Mannedorf, Switzerland).

### 4.12. Cholesteryl Ester Transfer Protein (CETP) Activity

CETP activity in serum was measured in duplicate using a commercially available kit (Merck, St. Louis, MO, USA) following the manufacturer’s instructions. Specifically, the assay uses a proprietary substrate to detect CETP-mediated transfer of neutral lipid from the substrate to its acceptor. The transfer activity results in an increase in fluorescence intensity at 465/535 nm. Measurements were obtained using a plate reader (Tecan Infinite 200 PRO) (Tecan, Mannedorf, Switzerland).

### 4.13. Lecithin–Cholesterol Acyltransferase (LCAT) Activity

LCAT activity in serum was measured in duplicate using a commercially available kit (Merck, St. Louis, MO, USA) following the manufacturer’s instructions. Specifically, samples were incubated with LCAT substrate for 3 h at 37 °C. The fluorescent substrate emits fluorescence at 470 nm. When the substrate is hydrolysed by LCAT, a monomer is released that emits fluorescence at 390 nm. The LCAT activity is expressed as a change in 470/390 nm emission intensity. Measurements were obtained using a plate reader (Tecan Infinite 200 PRO) (Tecan, Mannedorf, Switzerland).

### 4.14. Statistical Analysis

Data are shown as mean ± standard deviation unless otherwise specified. Normality was visually assessed from plots of the data, and logarithmic transformations were applied to skewed variables. “Post-Hoc” power analyses were performed with the freely available G*power software: all variables with an effect size (e.g., delta/sigma) > 1.1 were sufficiently powered and resulted in significant results. Comparisons of clinical and omics data between healthy and MetS were assessed using two-way ANOVA (with “sex” and “disease state” as covariates), and *p*-value < 0.05 was considered significant. Correlation matrices between omics layers and clinical variables were performed with Pearson correlation coefficients, with *p*-value < 0.05 considered significant, or multiple regression (lm function, in R) with “sex” as a covariate, when relevant; a *p*-value < 0.05 was considered significant. Log2 fold changes in the whole serum and HDL lipidome (volcano plots) were analysed by Student’s two-sided T-Test, with a *p*-value < 0.05 considered significant. The adjusted *p*-value calculated using the false discovery rate (FDR) was also calculated and is reported along with the unadjusted *p*-value in Supplementary Figures S2 and S5. Statistical analysis was performed with R version 4.0.0 and Microsoft Excel 2016. Graphs were obtained using R version 4.0.0 and Graph Pad (Graph Pad Prism 7.0, San Diego, CA, USA).

## Data Availability

The data that support the findings of this study are available on request from the corresponding authors [M.V., J.L.G.].

## Supplementary Materials

The following supporting information can be downloaded at: www.mdpi.com/article/10.3390/ijms23126786/s1.

## Abbreviations

apo	Apolipoprotein
BMI	Body mass index
CE	Cholesteryl esters
DG	Diglycerides
FC	Free cholesterol
FFA	Free fatty acids
HDL	High-density lipoprotein
HOMA2-IR	Homeostasis Model Assessment 2 of Insulin Resistance
LCAT	Lecithin–cholesterol acyltransferase
LDL	Low-density lipoprotein
LPC	Lysophosphatidylcholines
Lp-PLA2	Lipoprotein-associated phospholipase A2
MetS	Metabolic syndrome
SM	Sphingomyelins
PC	Phosphatidylcholines
TG	Triglycerides
