# Supplementary material for: Lipidomic Approaches to Study HDL Metabolism in Patients with Central Obesity Diagnosed with Metabolic Syndrome
Source: Int J Mol Sci. 2022 Jun 17;23(12):6786. doi: 10.3390/ijms23126786 (PMC9223701; doi:10.3390/ijms23126786)
Supplement: Supplementary file 1 [file ijms-23-06786-s001.zip › ijms-1697748-supplementary.pdf]

Lipidomic Approaches to Study HDL Metabolism in Patients with Central Obesity Diagnosed with Metabolic Syndrome

Gabriele Mocciaro [1,2,3], Simona D'Amore [4,5], Benjamin Jenkins [6], Richard Kay [6], Antonio Murgia [1], Luis Vicente Herrera-Marcos [7], Stefanie Neun [1], Alice P. Sowton [8], Zoe Hall [1,9], Susana Alejandra Palma-Duran [9], Giuseppe Palasciano [5], Frank Reimann [6], Andrew Murray [8], Patrizia Suppressa [2], Carlo Sabbà [2], Antonio Moschetta [2], Albert Koulman [6], Julian L. Griffin [1,9,10,*,†] and Michele Vacca [1,2,3,6,*,†]

[1] Department of Biochemistry and Cambridge Systems Biology Centre, University of Cambridge, Cambridge CB2 1GA, UK; gm593@cantab.ac.uk (G.M.); antonio.murgia@owlstone.co.uk (A.M.); sn504@cam.ac.uk (S.N.); zoe.hall@imperial.ac.uk (Z.H.)
[2] Department of Interdisciplinary Medicine, Clinica Medica "C. Frugoni", Aldo Moro University of Bari, Bari 70124, Italy; patrizia.suppressa@gmail.com (P.S.); carlo.sabba@uniba.it (C.S.); antonio.moschetta@uniba.it (A.M.)
[3] Roger Williams Institute of Hepatology, Foundation for Liver Research, London SE5 9NT, UK
[4] Department of Medicine, University of Cambridge, Cambridge CB2 0QQ, UK; simodamo@hotmail.it
[5] Clinica Medica "A. Murri", "Aldo Moro" University of Bari, Bari 70124, Italy; palascianog44@gmail.com
[6] Welcome Trust-MRC Institute of Metabolic Science Metabolic Research Laboratories, Addenbrooke's Hospital, Hills Road, Cambridge CB2 0QQ, UK; bjj25@medschl.cam.ac.uk (B.J.); rgk27@medschl.cam.ac.uk (R.K.); fr222@cam.ac.uk (F.R.); ak675@medschl.cam.ac.uk (A.K.)
[7] Department of Biochemistry and Molecular and Cellular Biology, Veterinary Faculty, University of Zaragoza, Zaragoza E-50013, Spain; luis.herrera@imdea.org
[8] Department of Physiology, Development and Neuroscience, University of Cambridge, Cambridge CB2 3EG, UK; apb72@cam.ac.uk (A.P.S.); ajm267@cam.ac.uk (A.M.)
[9] Biomolecular Medicine, Division of Systems Medicine, Department of Metabolism, Digestion and Reproduction, Imperial College London, London SW7 2AZ, UK; susana.palma-duran@crick.ac.uk
[10] Rowlett Institute, Foresterhill, University of Aberdeen, Aberdeen AB25 2ZD, UK
* Correspondence: jules.griffin@abdn.ac.uk (J.L.G.); michele.vacca@uniba.it (M.V.)
† These authors equally contributed to this work.

Supplementary materials

List of Supplementary Files:

1. Supplementary Figures 1-2

2. Supplementary Tables 1-5

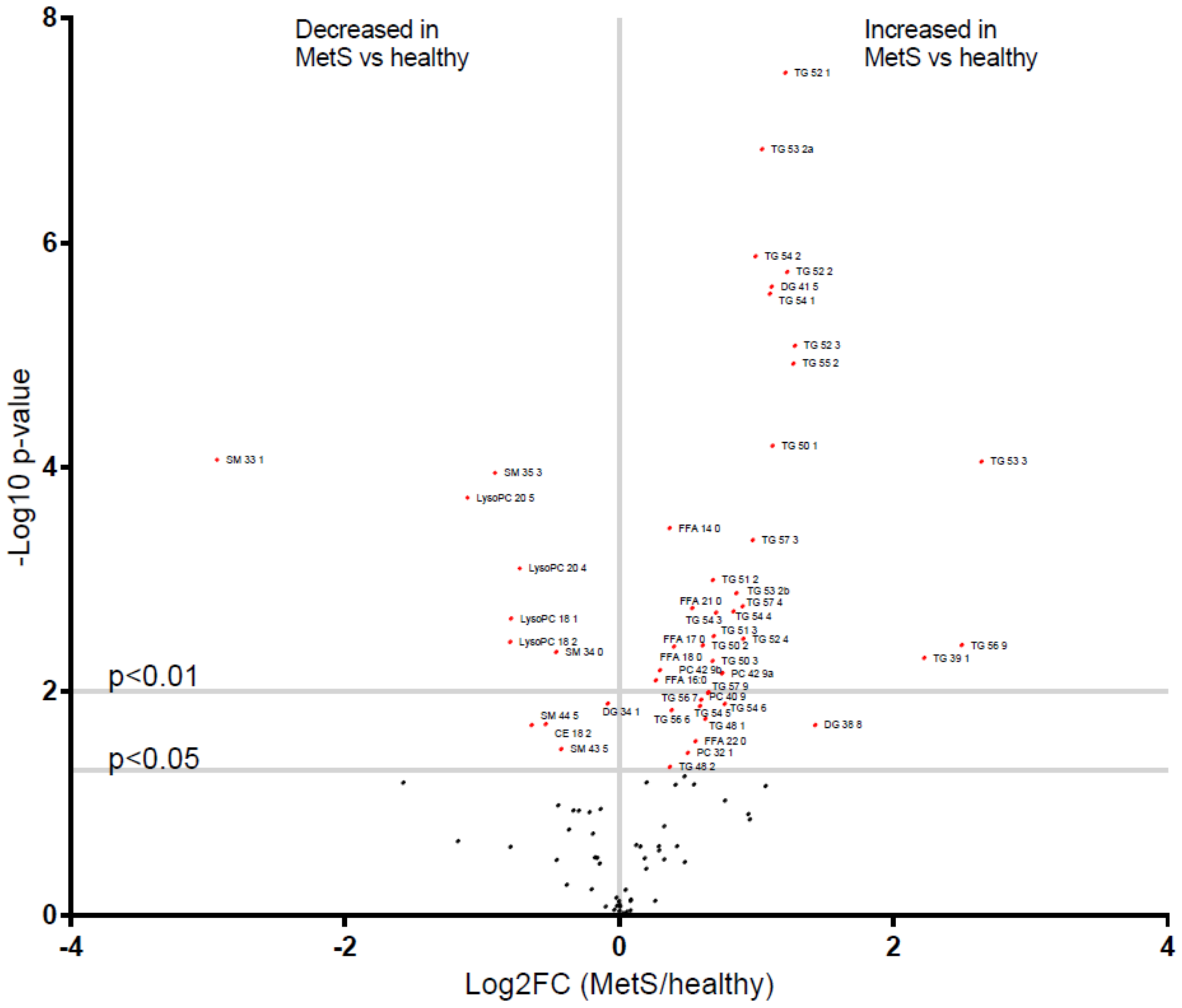

Figure S1. Volcano plot of whole serum lipidome. The whole serum lipidome of MetS participants (n = 14) showed lower levels of specific sphingomyelins (SM), lysophosphatidylcholines (LysoPC), cholesteryl esters (CE) and higher triglycerides (TG), diglycerides (DG) and some phosphatidylcholines (PC) when compared to healthy subjects (n = 11). Statistical significance was assessed by student two-sided T-Test. Lipid species were analysed by liquid chromatography-mass spectrometry as reported in the method section. Mean value of each lipid in healthy and MetS participants along with P-values and FDR are reported in Supplementary Table 4.

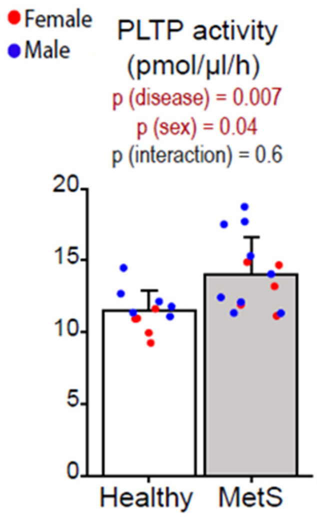

Figure S2. MetS is associated with higher PLTP activity. Phospholipid transfer protein (PLTP) activity was higher in the MetS group when compared with control. PLTP assays was performed on serum from healthy (*n* = 11) and MetS (*n* = 14) participants as detailed in the method section. Data are expressed as mean ± standard deviation. Statistical significance was assessed by two-way ANOVA using disease stage and sex as covariates; a *p*-value < 0.05 was considered significant.

Table S1. Clinical characteristics of the study cohort.

	Healthy	MetS	*p*-value	
			Sex	MetS
**N (M/F)**	6/5	9/5	-	0.6
**Age (years)**	29 ± 2	42 ± 10	0.4	**0.0004**
**Body mass index (Kg/m^2)**	22.8 ± 1.9	32.7 ± 3.4	0.1	**0.00000003**
**Waist circumference (cm)**	86 ± 8	111 ± 7	0.08	**0.00000004**
**SBP (mm/Hg)**	116 ± 8	122 ± 12	**0.02**	0.2
**DBP (mmHg)**	78 ± 6	80 ± 7	0.3	0.6
**Total cholesterol (mmol/L)**	5.5 ± 1	5 ± 1.1	0.2	0.4
**HDL-C (mmol/L)**	1.6 ± 0.4	0.9 ± 0.2	**0.001**	**0.000002**
**LDL-C (mmol/L)**	3.5 ± 0.9	3.4 ± 1.1	0.4	0.8
**Triglycerides (mmol/L)**	0.8 ± 0.3	1.5 ± 0.3	**0.003**	**0.00006**
**Glucose (mmol/L)**	5 ± 0.4	5.7 ± 0.7	0.7	**0.006**
**Insulin (pmol/L)**	53 ± 17	101 ± 61	0.4	**0.002**
**HOMA2-IR**	1 ± 0.3	1.9 ± 1.1	0.5	**0.01**
**ApoB-100/ApoA-1 ratio**	1.3 ± 0.3	2.1 ± 0.6	0.1	**0.002**

Data are expressed as mean ± standard deviation. Differences between the two groups were analysed with 2-way ANOVA using disease stage and sex as co-variates; a *p*-value < 0.05 was considered significant. Abbreviations: M = Male, F = Female, BMI = Body Mass Index, SBP=Systolic blood pressure, DBP=Diastolic blood pressure, HDL-C = high-density lipoprotein cholesterol, LDL-C = low-density lipoprotein cholesterol, TG = triglycerides, HOMA2-IR = Homeostasis Model Assessment 2 of Insulin Resistance; ApoB-100, apolipoprotein B-100; ApoA-1 apolipoprotein A-1.

Table S2. Average value for lipid species in the whole serum of Healthy and MetS participants.

Whole serum lipidome	Average normalised peak intensity - Healthy	Average normalised peak intensity - MetS	*p*-value	FDR
**CE 16 0**	0.411	0.402	0.884534653	0.930604166
**CE 18 0**	0.325	0.373	0.380575892	0.463110422
**CE 18 1**	39.352	39.596	0.832339241	0.884908035
**CE 18 2**	51.843	35.758	0.0196652	0.043010482
**CE 20 4**	24.528	19.485	0.115112903	0.189336767
**DG 32 0**	0.159321086	0.092046459	0.243699636	0.33261707
**DG 34 1**	0.108778231	0.102718975	0.012856743	0.031837802
**DG 37 0**	0.084824089	0.069177777	0.11622653	0.189336767
**DG 38 8**	0.349389706	0.940988321	0.020014779	0.043010482
**DG 41 5**	0.082156665	0.177741432	2.46519E-06	4.81E-05
**LysoPC 15 0**	0.010360738	0.007563991	0.319470554	0.403331574
**LysoPC 16 0**	0.692126414	0.693633619	0.909608664	0.937363362
**LysoPC 16 1**	0.014687905	0.015281642	0.918801711	0.937363362
**LysoPC 17 0**	0.012790905	0.011126363	0.580806068	0.690134268
**LysoPC 18 0**	0.325172269	0.252231062	0.170994451	0.257767755
**LysoPC 18 1**	0.269313457	0.156068212	0.002245602	0.00986112
**LysoPC 18 2**	0.268527176	0.154704396	0.003621153	0.013592192
**LysoPC 18 3**	0.140838234	0.149435702	0.720441127	0.823681686
**LysoPC 20 3**	0.063191507	0.046477716	0.104020014	0.178068159
**LysoPC 20 4**	0.043007377	0.026013381	0.000801808	0.005061411
**LysoPC 20 5**	0.052824818	0.024530703	0.000186948	0.001452442
**PC 30 0**	0.080767222	0.085586673	0.898560647	0.935614694
**PC 32 0**	0.281899667	0.313913895	0.242038273	0.33261707
**PC 32 1**	0.228358318	0.323204525	0.035343376	0.071393619
**PC 32 2**	0.062821669	0.083520648	0.068165627	0.122941577
**PC 34 1**	3.535585472	4.929156768	0.331956372	0.413920908
**PC 34 2**	6.568506053	9.593782425	0.067592892	0.122941577
**PC 34 4**	0.025174981	0.026685511	0.737229718	0.823681686
**PC 35 2**	0.138002281	0.142590461	0.590082186	0.693003497
**PC 36 4**	3.98809162	4.884301395	0.261416843	0.352041349
**PC 36 5**	0.308982022	0.388173434	0.314094329	0.401563635
**PC 36 6**	0.039237277	0.082340383	0.06977506	0.12363651
**PC 38 7**	0.57729321	0.774130529	0.241262138	0.33261707
**PC 38 8**	0.059159191	0.07102255	0.740415753	0.823681686
**PC 40 0**	0.08085624	0.155468724	0.124382921	0.196291797
**PC 40 5**	0.093837589	0.160207878	0.094168001	0.163982209
**PC 40 9**	0.271965242	0.409207094	0.013470016	0.032392181
**PC 42 8**	0.026458309	0.032343396	0.239925001	0.33261707
**PC 42 9a**	0.064336461	0.108317521	0.006892067	0.019888537
**PC 42 9b**	0.064336461	0.108317521	0.006892067	0.019888537
**SM 26 1**	0.038403762	0.07439313	0.138833026	0.215725163
**SM 32 1**	0.093972905	0.092958474	0.816508278	0.884908035
**SM 33 1**	0.31081373	0.040715321	8.61905E-05	0.000818946
**SM 34 0**	0.044982104	0.03273963	0.00446445	0.015030316
**SM 34 2**	0.128144362	0.114845383	0.304990085	0.397366428
**SM 34 4**	0.060446233	0.026742823	0.217432725	0.318271091
**SM 35 3**	0.02886031	0.015415948	0.000113006	0.000951138
**SM 35 4**	0.054145167	0.018189352	0.065268148	0.122075611
**SM 36 2**	0.080053791	0.080049914	0.742129044	0.823681686
**SM 36 4**	0.359171212	0.36641058	0.947735205	0.957212557
**SM 39 1**	0.046499889	0.042180325	0.343569225	0.423176728
**SM 40 1**	0.45293963	0.630525155	0.057244268	0.111185981
**SM 41 1**	0.162961689	0.144313469	0.302269104	0.397366428
**SM 41 2**	0.076223142	0.080969042	0.976977997	0.976977997
**SM 42 2**	0.702713625	0.615523888	0.185853935	0.276047757

**SM 42 4**	0.157507504	0.143491559	0.111666428	0.18797182
**SM 43 5**	0.034128588	0.025486603	0.032657117	0.06731365
**SM 44 4**	0.114987985	0.099041189	0.119949413	0.192299852
**SM 44 5**	0.367182916	0.235831394	0.019932648	0.043010482
**TG 39 1**	0.278069549	1.300649016	0.005025656	0.016373912
**TG 46 1**	0.119341106	0.13000335	0.234485553	0.33261707
**TG 48 1**	0.394532932	0.610449585	0.017553444	0.040293133
**TG 48 2**	0.286131151	0.369823345	0.047246376	0.093566353
**TG 50 1**	0.836468903	1.815968634	6.45962E-05	0.000724912
**TG 50 2**	1.248247296	1.905620078	0.003902709	0.013592192
**TG 50 3**	0.495514687	0.794676077	0.005346223	0.016874017
**TG 51 2**	0.129254553	0.207446742	0.001014893	0.006029658
**TG 51 3**	0.076587605	0.123505963	0.003224022	0.01356776
**TG 52 1**	0.61358433	1.421168547	3.04712E-08	3.08E-06
**TG 52 2**	2.600571787	6.08536918	1.82628E-06	4.61E-05
**TG 52 3**	2.075300893	5.046089209	8.26253E-06	0.000119217
**TG 52 4**	0.950946332	1.782204882	0.003397666	0.013592192
**TG 53 2 a**	0.075540129	0.155602421	1.46336E-07	7.39E-06
**TG 53 2 b**	0.075760345	0.137174887	0.001333268	0.007481114
**TG 53 3**	0.081434785	0.508221769	8.91922E-05	0.000818946
**TG 54 1**	0.114106124	0.244500483	2.85585E-06	4.81E-05
**TG 54 2**	0.346611382	0.69036496	1.31754E-06	4.44E-05
**TG 54 3**	1.105323274	1.805550841	0.001989238	0.009132412
**TG 54 4**	0.898778359	1.602380526	0.00193844	0.009132412
**TG 54 5**	0.398020176	0.678646158	0.012924256	0.031837802
**TG 54 6**	0.143875205	0.217944487	0.011898269	0.030813465
**TG 55 2**	0.17100972	0.412848174	1.19937E-05	0.000151421
**TG 56 6**	0.219177678	0.286022492	0.014695336	0.034516952
**TG 56 7**	0.135282204	0.212061272	0.010284861	0.027336077
**TG 56 9**	0.261915077	1.481717308	0.00385904	0.013592192
**TG 57 3**	0.054153722	0.106533008	0.000447025	0.003009969
**TG 57 4**	0.048980741	0.091410204	0.001743356	0.009110976
**TG 57 9**	0.034622606	0.054390902	0.010198682	0.027336077
**FFA 14 0**	0.302	0.389	0.000351032	0.002532448
**FFA 15 0**	0.035	0.040	0.064928655	0.122075611
**FFA 16 0**	7.142106257	8.600472092	0.00797862	0.022384463
**FFA 17 0**	0.074683697	0.095778543	0.003683136	0.013592192
**FFA 17 1**	0.035861929	0.035933378	0.795237938	0.873032954
**FFA 18 0**	6.853645383	8.433201254	0.006469691	0.019801175
**FFA 18 1**	1.06005879	1.331500654	0.159480253	0.244053115
**FFA 18 2**	0.270751664	0.30841671	0.306877044	0.397366428
**FFA 18 3**	0.07722938	0.059335179	0.529180317	0.636276333
**FFA 20 4**	0.092097111	0.086052748	0.827656182	0.884908035
**FFA 21 0**	0.012456402	0.018018219	0.001804154	0.009110976
**FFA 22 0**	0.012527612	0.018430634	0.027877688	0.058659302
**FFA 22 6**	0.026136606	0.025782511	0.691882355	0.803219745

Statistical significance was calculated using a two-tailed Student-T test on log2-transformed lipids; the adjusted *p*-value was calculated using the false discovery rate (FDR) as described in the methods.

Table S3. Targeted (LC MS/MS) parameters for the measurements of circulating cholesteryl esters (CE).

Metabolite	HMDB ID	Column	Retention Time (RT)	Mode	Precursor	Product	Collision Energy (CE)	Lens
			(min)		*m/z*	*m/z*	(V)	(V)
CE15:0 d7		18 CSH	7.6	Positive	640.58	376.387	24.713	155
CE16:0	HMDB0000885	18 CSH	7.7	Positive	647.815	369.333	24.511	140
CE18:1	HMDB0005189	18 CSH	7.6	Positive	673.537	369.333	27.242	152
CE18:2	HMDB0000610	18 CSH	7.5	Positive	671.53	369.333	28.86	151
CE18:3	HMDB0010369	18 CSH	7.2	Positive	669.56	369.333	21	151
CE 20:4	HMDB0006726	18 CSH	7.4	Positive	695.567	369.333	30.124	148

Table S4. Targeted (LC MS/MS) parameters for the measurements of circulating apolipoproteins.

Protein	Peptide sequence	Q1 *m/z*	Q3 *m/z*	Collision Energy (CE)
APOA-I	DYVSQFEGSALGK	700.8	808.4	20
APOA-IV	LGEVNTYAGDLQK	675.7	540.2	20
		675.7	575.6	20
APOB-100	TEVIPPLIENR	640.8	838.4	20
APOC-I	TPDVSSALDK	516.8	620.3	20
		516.8	719.4	20
APOC-II	TYLPAVDEK	518.2	658.2	15
		518.2	771.2	15
APOC-III	GWVTDGFSSLK	598.7	854.2	20
		598.7	953.3	20
APOD	NILTSNNIDVK	615.8	890.4	20
		615.8	1003.5	20
APOE	LGPLVEQGR	484.7	399.7	15
		484.7	588.2	20
APOM	EFPEVHLGQWYFIAGAAP TK	754.4	615.8	30
BSA 1	LVNELTEFAK	582.3	951.29	20
BSA 2	LGEYGFQNALIVR	740.5	1017.4	25

Table S5. Average value for lipid species in the HDL of Healthy and MetS participants.

HDL lipidome	Average normalised peak intensity Healthy	Average normalised peak intensity MetS	*p*-value	FDR
CE 16 0	0.338540925	0.378028828	0.623829276	0.836281708
CE 18 1	16.21745037	11.25866694	0.052599514	0.173383585
CE 18 2	204.7446748	124.1649671	0.011707837	0.148856784
CE 20 4	80.78008219	61.23188783	0.231031364	0.489566461
LysoPC 16 0	0.820194003	0.794781016	0.855615207	0.961997015
LysoPC 16 1	0.004558758	0.003564052	0.35580107	0.620907749
LysoPC 17 0	0.021753407	0.021389974	0.967406142	0.983175306
LysoPC 18 0	0.570777088	0.580768708	0.775485956	0.932679055
LysoPC 18 1	0.234170864	0.214919709	0.649309269	0.837514854
LysoPC 18 2	0.129858771	0.083476155	0.031566421	0.158588981
LysoPC 18 3	0.200119696	0.189048574	0.720528086	0.890149461
LysoPC 20 3	0.144288832	0.139153262	0.981574291	0.983175306
LysoPC 20 4	0.081315063	0.071639247	0.50132205	0.769270042
LysoPC 20 5	0.0445405	0.029561901	0.050927484	0.173383585
PC 30 0	0.007740762	0.005115792	0.022315515	0.158588981
PC 30 1	0.001444872	0.001196408	0.606519372	0.836281708
PC 31 0	0.004614647	0.00178596	0.000152811	0.013600221
PC 32 0	0.092930064	0.063645056	0.004252601	0.064803468
PC 32 1	0.061851449	0.060558	0.983175306	0.983175306
PC 32 2	0.016996051	0.015416545	0.634268055	0.836281708
PC 33 0	0.00242063	0.001368813	0.022615386	0.158588981
PC 33 1	0.016056487	0.01289907	0.204304112	0.476465335
PC 33 2	0.031933493	0.023471885	0.042550242	0.158588981
PC 34 0	0.010579054	0.007266578	0.003270763	0.064803468
PC 34 1	2.034745873	1.73082767	0.164358477	0.39534877
PC 34 2	3.476053076	2.747097559	0.043013657	0.158588981
PC 34 3	0.082776371	0.076997158	0.665160513	0.838107651
PC 34 4	0.00462267	0.00455171	0.833586244	0.961997015
PC 35 1	0.024583278	0.019087934	0.100082534	0.276368772
PC 35 2	0.056418545	0.042990834	0.065223441	0.200168493
PC 35 3	0.005983456	0.005150018	0.496168175	0.769270042
PC 35 4	0.010493651	0.00865644	0.214856862	0.476465335
PC 36 1	0.187948922	0.145242462	0.089912285	0.266739778
PC 36 2	1.277281463	1.050659014	0.042284199	0.158588981
PC 36 3	0.812382053	0.721558791	0.292291274	0.553487732
PC 36 4	1.049740008	0.983048569	0.575740707	0.813347983
PC 37 3	0.004972606	0.004514469	0.386492592	0.658878302
PC 37 4	0.013861205	0.011413757	0.320410169	0.584942402
PC 37 5	0.003166099	0.003569474	0.828546422	0.961997015
PC 37 6	0.002658056	0.002709181	0.979677137	0.983175306
PC 38 2	0.024695643	0.02185884	0.430808477	0.710036194
PC 38 3	0.169732144	0.201896659	0.211208686	0.476465335
PC 38 4	0.74189748	0.695025313	0.552595888	0.806246459
PC 38 5	0.287121725	0.258353072	0.471846351	0.749898665
PC 38 6	0.434175289	0.454369	0.824929228	0.961997015
PC 39 6	0.001636547	0.002197552	0.63895681	0.836281708
PC 40 4	0.008522435	0.008554048	0.930665677	0.974461709
PC 40 5	0.027763257	0.027303223	0.922826934	0.974461709
PC 40 6	0.116564651	0.13085137	0.538332908	0.80490238
PC 40 7	0.015644849	0.015560681	0.883331027	0.961997015
PC 28 0	0.00109182	0.0006912	0.039453749	0.158588981
SM 32 0	0.000302537	0.000232601	0.219495267	0.476465335
SM 32 1	0.031888249	0.02608093	0.032467514	0.158588981
SM 33 0	0.002429473	0.00262276	0.61401929	0.836281708

**SM 33 1**	0.014296825	0.010314505	0.004368773	0.064803468
**SM 34 0**	0.013058918	0.008003651	0.002458824	0.064803468
**SM 34 1**	0.626756539	0.425283243	0.001295217	0.057637155
**SM 34 2**	0.045148687	0.038144289	0.105579082	0.276368772
**SM 35 1**	0.010497086	0.007179481	0.015528282	0.153557454
**SM 35 2**	0.001136128	0.000937003	0.249748889	0.51692212
**SM 36 1**	0.082790498	0.065728927	0.099909629	0.276368772
**SM 36 2**	0.030235324	0.02608769	0.392365731	0.658878302
**SM 36 3**	0.001721493	0.00152746	0.565597702	0.811906379
**SM 38 1**	0.03565122	0.026333007	0.019850458	0.158588981
**SM 38 2**	0.030428857	0.02204447	0.028838667	0.158588981
**SM 39 1**	0.010290486	0.007721538	0.023973192	0.158588981
**SM 40 0**	0.000608204	0.000910179	0.34793977	0.619332791
**SM 40 1**	0.068845588	0.053772464	0.03351592	0.158588981
**SM 40 2**	0.064655861	0.048295186	0.013966115	0.153557454
**SM 41 1**	0.02287977	0.018455367	0.117246209	0.29814036
**SM 42 1**	0.049868766	0.03715174	0.040277282	0.158588981
**SM 42 2**	0.123749041	0.089865611	0.029189626	0.158588981
**SM 46 0**	0.001022669	0.00123924	0.103099405	0.276368772
**SM 48 0**	0.013629228	0.016802189	0.044547466	0.158588981
**SM 30 1**	0.002553294	0.002131579	0.32204694	0.584942402
**TG 12 0**	0.017840687	0.021320926	0.292154587	0.553487732
**TG 18 0**	0.002882784	0.003778043	0.886334328	0.961997015
**TG 52 3**	0.131747512	0.172092386	0.042095639	0.158588981
**TG 52 4**	0.050505198	0.063372905	0.12098313	0.299097182
**TG 56 6**	0.005339102	0.005903429	0.454233978	0.735033164
**TG 56 7**	0.00542369	0.007335577	0.266326986	0.526735595
**TG 58 10**	0.00158219	0.001589258	0.867994033	0.961997015
**TG 58 12**	0.000571951	0.000619179	0.730122592	0.890149461
**TG 58 7**	0.004268805	0.00472811	0.542630818	0.80490238
**TG 59 8**	0.001742913	0.002032943	0.261536373	0.526735595
**TG 59 9**	0.001496814	0.002174335	0.913253861	0.974461709

Statistical significance was calculated using a two-tailed Student-T test on log2-transformed lipids; the adjusted *p*-value was calculated using the false discovery rate (FDR) as described in the methods.
